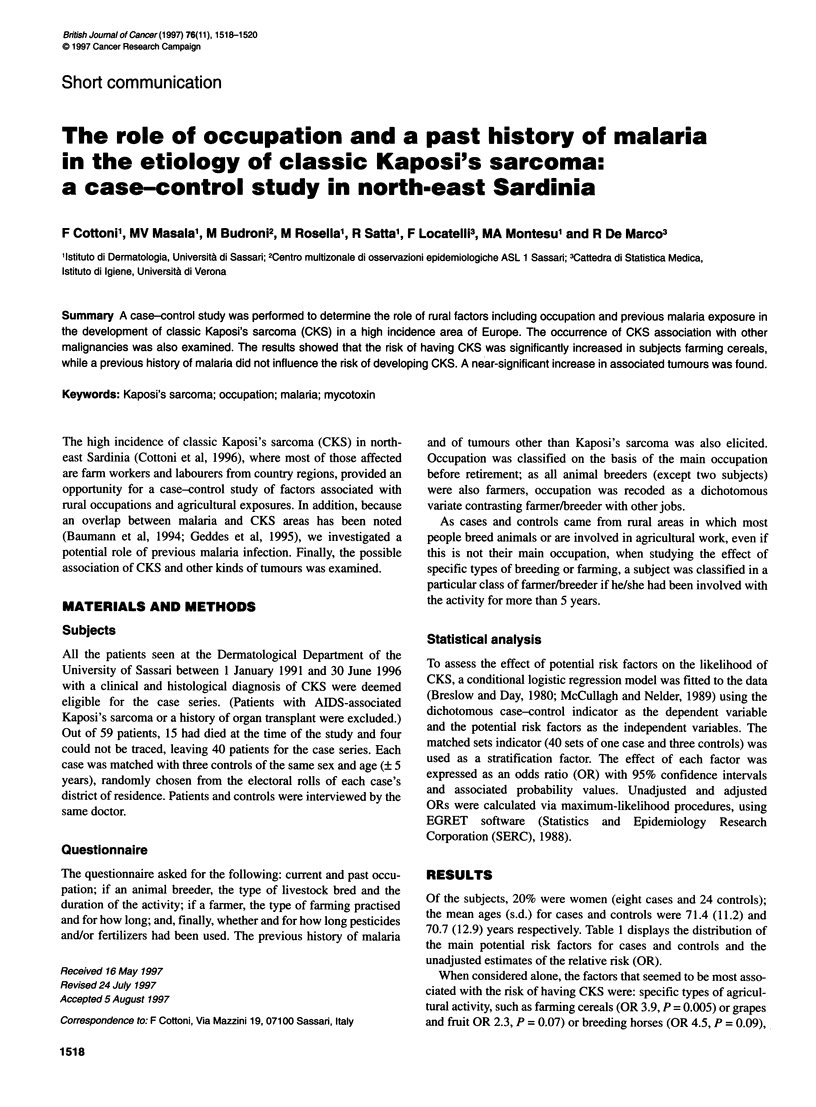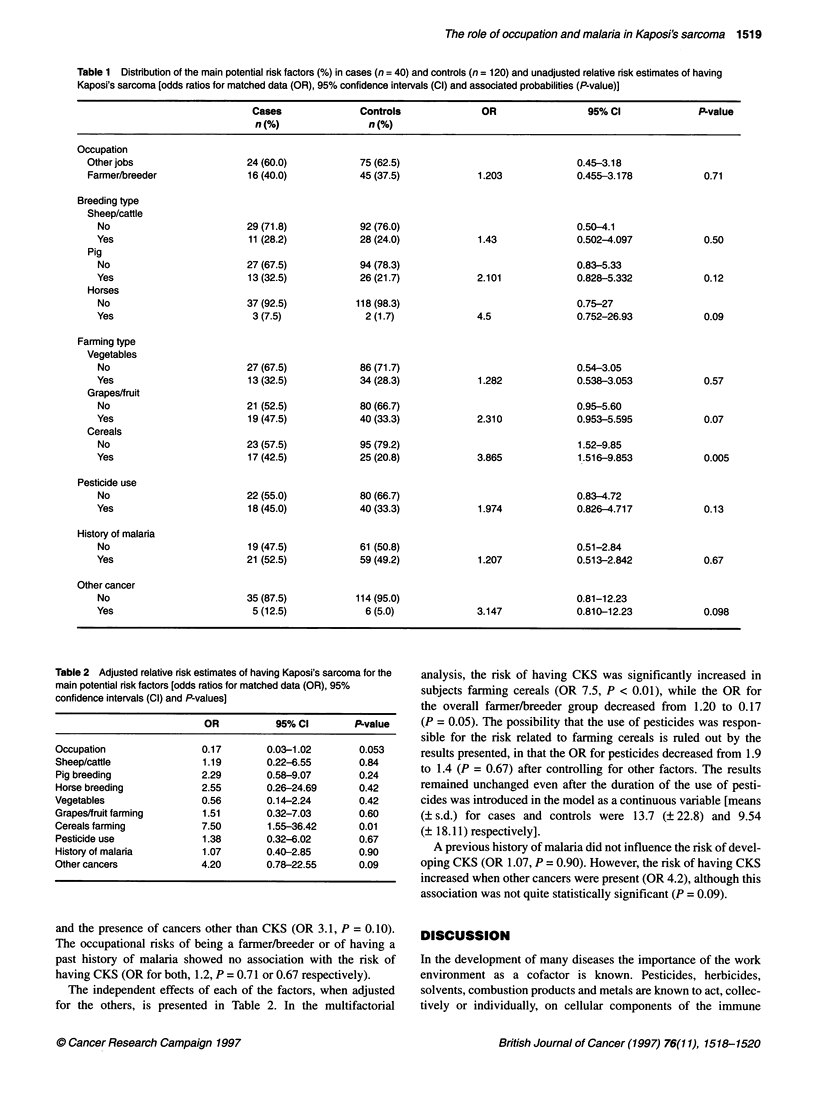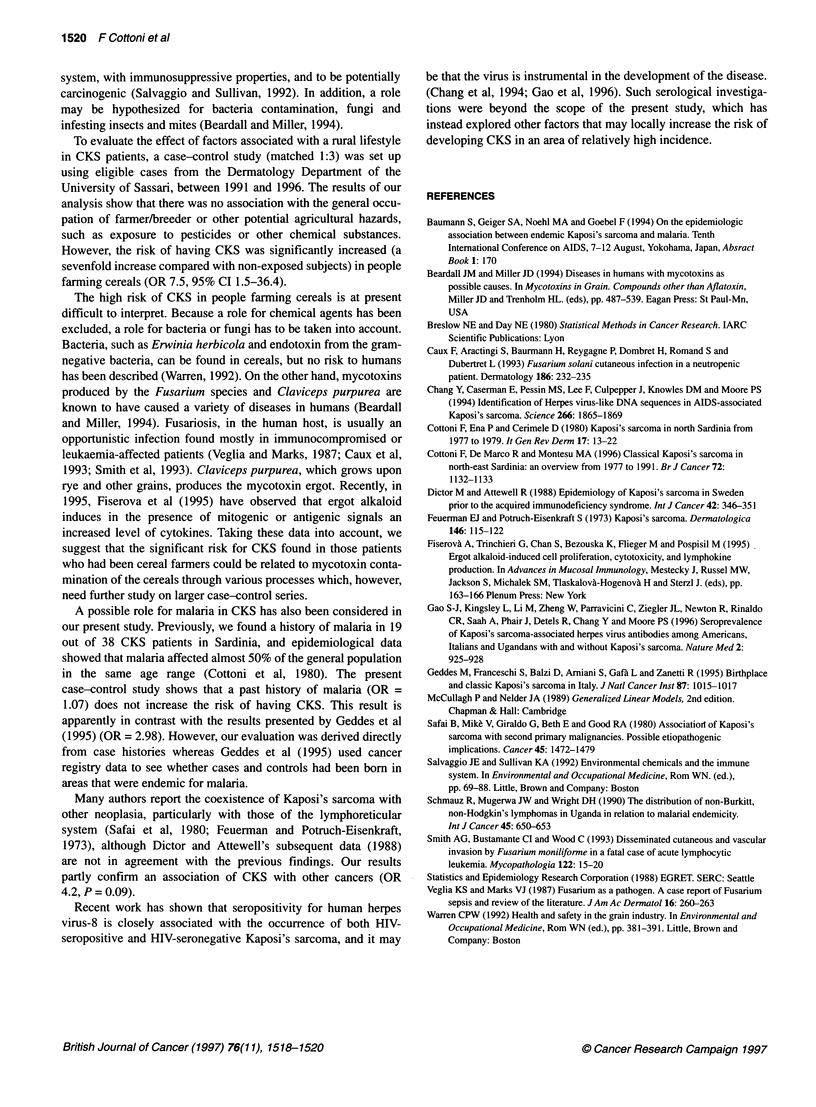# The role of occupation and a past history of malaria in the etiology of classic Kaposi's sarcoma: a case-control study in north-east Sardinia.

**DOI:** 10.1038/bjc.1997.587

**Published:** 1997

**Authors:** F. Cottoni, M. V. Masala, M. Budroni, M. Rosella, R. Satta, F. Locatelli, M. A. Montesu, R. De Marco

**Affiliations:** Istituto di Dermatologia, UniversitÃ di Sassari, Italy.

## Abstract

A case-control study was performed to determine the role of rural factors including occupation and previous malaria exposure in the development of classic Kaposi's sarcoma (CKS) in a high incidence area of Europe. The occurrence of CKS association with other malignancies was also examined. The results showed that the risk of having CKS was significantly increased in subjects farming cereals, while a previous history of malaria did not influence the risk of developing CKS. A near-significant increase in associated tumours was found.


British Journal of Cancer (1997) 76(11), 1518–1520
© 1997 Cancer Research Campaign

Short communication

### The role of occupation and a past history of malaria in the etiology of classic Kaposi's sarcoma: a case–control study in north-east Sardinia

**F Cottoni[1], MV Masala[1], M Budroni[2], M Rosella[1], R Satta[1], F Locatelli[3], MA Montesu[1] and R De Marco[3]**

[1]Istituto di Dermatologia, Università di Sassari; [2]Centro multizonale di osservazioni epidemiologiche ASL 1 Sassari; [3]Cattedra di Statistica Medica, Istituto di Igiene, Università di Verona

**Summary** A case–control study was performed to determine the role of rural factors including occupation and previous malaria exposure in the development of classic Kaposi's sarcoma (CKS) in a high incidence area of Europe. The occurrence of CKS association with other malignancies was also examined. The results showed that the risk of having CKS was significantly increased in subjects farming cereals, while a previous history of malaria did not influence the risk of developing CKS. A near-significant increase in associated tumours was found.

**Keywords:** Kaposi's sarcoma; occupation; malaria; mycotoxin

The high incidence of classic Kaposi's sarcoma (CKS) in north-east Sardinia (Cottoni et al, 1996), where most of those affected are farm workers and labourers from country regions, provided an opportunity for a case–control study of factors associated with rural occupations and agricultural exposures. In addition, because an overlap between malaria and CKS areas has been noted (Baumann et al, 1994; Geddes et al, 1995), we investigated a potential role of previous malaria infection. Finally, the possible association of CKS and other kinds of tumours was examined.

### MATERIALS AND METHODS

### Subjects

All the patients seen at the Dermatological Department of the University of Sassari between 1 January 1991 and 30 June 1996 with a clinical and histological diagnosis of CKS were deemed eligible for the case series. (Patients with AIDS-associated Kaposi's sarcoma or a history of organ transplant were excluded.) Out of 59 patients, 15 had died at the time of the study and four could not be traced, leaving 40 patients for the case series. Each case was matched with three controls of the same sex and age (± 5 years), randomly chosen from the electoral rolls of each case's district of residence. Patients and controls were interviewed by the same doctor.

### Questionnaire

The questionnaire asked for the following: current and past occupation; if an animal breeder, the type of livestock bred and the duration of the activity; if a farmer, the type of farming practised and for how long; and, finally, whether and for how long pesticides and/or fertilizers had been used. The previous history of malaria and of tumours other than Kaposi's sarcoma was also elicited. Occupation was classified on the basis of the main occupation before retirement; as all animal breeders (except two subjects) were also farmers, occupation was recoded as a dichotomous variate contrasting farmer/breeder with other jobs.

As cases and controls came from rural areas in which most people breed animals or are involved in agricultural work, even if this is not their main occupation, when studying the effect of specific types of breeding or farming, a subject was classified in a particular class of farmer/breeder if he/she had been involved with the activity for more than 5 years.

### Statistical analysis

To assess the effect of potential risk factors on the likelihood of CKS, a conditional logistic regression model was fitted to the data (Breslow and Day, 1980; McCullagh and Nelder, 1989) using the dichotomous case–control indicator as the dependent variable and the potential risk factors as the independent variables. The matched sets indicator (40 sets of one case and three controls) was used as a stratification factor. The effect of each factor was expressed as an odds ratio (OR) with 95% confidence intervals and associated probability values. Unadjusted and adjusted ORs were calculated via maximum-likelihood procedures, using EGRET software (Statistics and Epidemiology Research Corporation (SERC), 1988).

### RESULTS

Of the subjects, 20% were women (eight cases and 24 controls); the mean ages (s.d.) for cases and controls were 71.4 (11.2) and 70.7 (12.9) years respectively. Table 1 displays the distribution of the main potential risk factors for cases and controls and the unadjusted estimates of the relative risk (OR).

When considered alone, the factors that seemed to be most associated with the risk of having CKS were: specific types of agricultural activity, such as farming cereals (OR 3.9, $P = 0.005$) or grapes and fruit OR 2.3, $P = 0.07$) or breeding horses (OR 4.5, $P = 0.09$),

*Received 16 May 1997*
*Revised 24 July 1997*
*Accepted 5 August 1997*

*Correspondence to:* F Cottoni, Via Mazzini 19, 07100 Sassari, Italy

**Table 1** Distribution of the main potential risk factors (%) in cases (*n* = 40) and controls (*n* = 120) and unadjusted relative risk estimates of having Kaposi's sarcoma [odds ratios for matched data (OR), 95% confidence intervals (CI) and associated probabilities (*P*-value)]

| | Cases *n* (%) | Controls *n* (%) | OR | 95% CI | *P*-value |
|---|---|---|---|---|---|
| Occupation | | | | | |
| Other jobs | 24 (60.0) | 75 (62.5) | | 0.45–3.18 | |
| Farmer/breeder | 16 (40.0) | 45 (37.5) | 1.203 | 0.455–3.178 | 0.71 |
| Breeding type | | | | | |
| Sheep/cattle | | | | | |
| No | 29 (71.8) | 92 (76.0) | | 0.50–4.1 | |
| Yes | 11 (28.2) | 28 (24.0) | 1.43 | 0.502–4.097 | 0.50 |
| Pig | | | | | |
| No | 27 (67.5) | 94 (78.3) | | 0.83–5.33 | |
| Yes | 13 (32.5) | 26 (21.7) | 2.101 | 0.828–5.332 | 0.12 |
| Horses | | | | | |
| No | 37 (92.5) | 118 (98.3) | | 0.75–27 | |
| Yes | 3 (7.5) | 2 (1.7) | 4.5 | 0.752–26.93 | 0.09 |
| Farming type | | | | | |
| Vegetables | | | | | |
| No | 27 (67.5) | 86 (71.7) | | 0.54–3.05 | |
| Yes | 13 (32.5) | 34 (28.3) | 1.282 | 0.538–3.053 | 0.57 |
| Grapes/fruit | | | | | |
| No | 21 (52.5) | 80 (66.7) | | 0.95–5.60 | |
| Yes | 19 (47.5) | 40 (33.3) | 2.310 | 0.953–5.595 | 0.07 |
| Cereals | | | | | |
| No | 23 (57.5) | 95 (79.2) | | 1.52–9.85 | |
| Yes | 17 (42.5) | 25 (20.8) | 3.865 | 1.516–9.853 | 0.005 |
| Pesticide use | | | | | |
| No | 22 (55.0) | 80 (66.7) | | 0.83–4.72 | |
| Yes | 18 (45.0) | 40 (33.3) | 1.974 | 0.826–4.717 | 0.13 |
| History of malaria | | | | | |
| No | 19 (47.5) | 61 (50.8) | | 0.51–2.84 | |
| Yes | 21 (52.5) | 59 (49.2) | 1.207 | 0.513–2.842 | 0.67 |
| Other cancer | | | | | |
| No | 35 (87.5) | 114 (95.0) | | 0.81–12.23 | |
| Yes | 5 (12.5) | 6 (5.0) | 3.147 | 0.810–12.23 | 0.098 |

**Table 2** Adjusted relative risk estimates of having Kaposi's sarcoma for the main potential risk factors [odds ratios for matched data (OR), 95% confidence intervals (CI) and *P*-values]

| | OR | 95% CI | *P*-value |
|---|---|---|---|
| Occupation | 0.17 | 0.03–1.02 | 0.053 |
| Sheep/cattle | 1.19 | 0.22–6.55 | 0.84 |
| Pig breeding | 2.29 | 0.58–9.07 | 0.24 |
| Horse breeding | 2.55 | 0.26–24.69 | 0.42 |
| Vegetables | 0.56 | 0.14–2.24 | 0.42 |
| Grapes/fruit farming | 1.51 | 0.32–7.03 | 0.60 |
| Cereals farming | 7.50 | 1.55–36.42 | 0.01 |
| Pesticide use | 1.38 | 0.32–6.02 | 0.67 |
| History of malaria | 1.07 | 0.40–2.85 | 0.90 |
| Other cancers | 4.20 | 0.78–22.55 | 0.09 |

and the presence of cancers other than CKS (OR 3.1, $P = 0.10$). The occupational risks of being a farmer/breeder or of having a past history of malaria showed no association with the risk of having CKS (OR for both, 1.2, $P = 0.71$ or 0.67 respectively).

The independent effects of each of the factors, when adjusted for the others, is presented in Table 2. In the multifactorial analysis, the risk of having CKS was significantly increased in subjects farming cereals (OR 7.5, $P < 0.01$), while the OR for the overall farmer/breeder group decreased from 1.20 to 0.17 ($P = 0.05$). The possibility that the use of pesticides was responsible for the risk related to farming cereals is ruled out by the results presented, in that the OR for pesticides decreased from 1.9 to 1.4 ($P = 0.67$) after controlling for other factors. The results remained unchanged even after the duration of the use of pesticides was introduced in the model as a continuous variable [means (± s.d.) for cases and controls were 13.7 (± 22.8) and 9.54 (± 18.11) respectively].

A previous history of malaria did not influence the risk of developing CKS (OR 1.07, $P = 0.90$). However, the risk of having CKS increased when other cancers were present (OR 4.2), although this association was not quite statistically significant ($P = 0.09$).

### DISCUSSION

In the development of many diseases the importance of the work environment as a cofactor is known. Pesticides, herbicides, solvents, combustion products and metals are known to act, collectively or individually, on cellular components of the immune system, with immunosuppressive properties, and to be potentially carcinogenic (Salvaggio and Sullivan, 1992). In addition, a role may be hypothesized for bacteria contamination, fungi and infesting insects and mites (Beardall and Miller, 1994).

To evaluate the effect of factors associated with a rural lifestyle in CKS patients, a case–control study (matched 1:3) was set up using eligible cases from the Dermatology Department of the University of Sassari, between 1991 and 1996. The results of our analysis show that there was no association with the general occupation of farmer/breeder or other potential agricultural hazards, such as exposure to pesticides or other chemical substances. However, the risk of having CKS was significantly increased (a sevenfold increase compared with non-exposed subjects) in people farming cereals (OR 7.5, 95% CI 1.5–36.4).

The high risk of CKS in people farming cereals is at present difficult to interpret. Because a role for chemical agents has been excluded, a role for bacteria or fungi has to be taken into account. Bacteria, such as *Erwinia herbicola* and endotoxin from the gram-negative bacteria, can be found in cereals, but no risk to humans has been described (Warren, 1992). On the other hand, mycotoxins produced by the *Fusarium* species and *Claviceps purpurea* are known to have caused a variety of diseases in humans (Beardall and Miller, 1994). Fusariosis, in the human host, is usually an opportunistic infection found mostly in immunocompromised or leukaemia-affected patients (Veglia and Marks, 1987; Caux et al, 1993; Smith et al, 1993). *Claviceps purpurea*, which grows upon rye and other grains, produces the mycotoxin ergot. Recently, in 1995, Fiserova et al (1995) have observed that ergot alkaloid induces in the presence of mitogenic or antigenic signals an increased level of cytokines. Taking these data into account, we suggest that the significant risk for CKS found in those patients who had been cereal farmers could be related to mycotoxin contamination of the cereals through various processes which, however, need further study on larger case–control series.

A possible role for malaria in CKS has also been considered in our present study. Previously, we found a history of malaria in 19 out of 38 CKS patients in Sardinia, and epidemiological data showed that malaria affected almost 50% of the general population in the same age range (Cottoni et al, 1980). The present case–control study shows that a past history of malaria (OR = 1.07) does not increase the risk of having CKS. This result is apparently in contrast with the results presented by Geddes et al (1995) (OR = 2.98). However, our evaluation was derived directly from case histories whereas Geddes et al (1995) used cancer registry data to see whether cases and controls had been born in areas that were endemic for malaria.

Many authors report the coexistence of Kaposi's sarcoma with other neoplasia, particularly with those of the lymphoreticular system (Safai et al, 1980; Feuerman and Potruch-Eisenkraft, 1973), although Dictor and Attewell's subsequent data (1988) are not in agreement with the previous findings. Our results partly confirm an association of CKS with other cancers (OR 4.2, $P = 0.09$).

Recent work has shown that seropositivity for human herpes virus-8 is closely associated with the occurrence of both HIV-seropositive and HIV-seronegative Kaposi's sarcoma, and it may be that the virus is instrumental in the development of the disease. (Chang et al, 1994; Gao et al, 1996). Such serological investigations were beyond the scope of the present study, which has instead explored other factors that may locally increase the risk of developing CKS in an area of relatively high incidence.

### REFERENCES

Baumann S, Geiger SA, Noehl MA and Goebel F (1994) On the epidemiologic association between endemic Kaposi's sarcoma and malaria. Tenth International Conference on AIDS, 7–12 August, Yokohama, Japan, *Absract Book* **1**: 170

Beardall JM and Miller JD (1994) Diseases in humans with mycotoxins as possible causes. In *Mycotoxins in Grain. Compounds other than Aflatoxin*, Miller JD and Trenholm HL. (eds), pp. 487–539. Eagan Press: St Paul-Mn, USA

Breslow NE and Day NE (1980) *Statistical Methods in Cancer Research.* IARC Scientific Publications: Lyon

Caux F, Aractingi S, Baurmann H, Reygagne P, Dombret H, Romand S and Dubertret L (1993) *Fusarium solani* cutaneous infection in a neutropenic patient. Dermatology **186**: 232–235

Chang Y, Caserman E, Pessin MS, Lee F, Culpepper J, Knowles DM and Moore PS (1994) Identification of Herpes virus-like DNA sequences in AIDS-associated Kaposi's sarcoma. *Science* **266**: 1865–1869

Cottoni F, Ena P and Cerimele D (1980) Kaposi's sarcoma in north Sardinia from 1977 to 1979. *It Gen Rev Derm* **17**: 13–22

Cottoni F, De Marco R and Montesu MA (1996) Classical Kaposi's sarcoma in north-east Sardinia: an overview from 1977 to 1991. *Br J Cancer* **72**: 1132–1133

Dictor M and Attewell R (1988) Epidemiology of Kaposi's sarcoma in Sweden prior to the acquired immunodeficiency syndrome. *Int J Cancer* **42**: 346–351

Feuerman EJ and Potruch-Eisenkraft S (1973) Kaposi's sarcoma. *Dermatologica* **146**: 115–122

Fiserovà A, Trinchieri G, Chan S, Bezouska K, Flieger M and Pospisil M (1995) Ergot alkaloid-induced cell proliferation, cytotoxicity, and lymphokine production. In *Advances in Mucosal Immunology*, Mestecky J, Russel MW, Jackson S, Michalek SM, Tlaskalovà-Hogenovà H and Sterzl J. (eds), pp. 163–166 Plenum Press: New York

Gao S-J, Kingsley L, Li M, Zheng W, Parravicini C, Ziegler JL, Newton R, Rinaldo CR, Saah A, Phair J, Detels R, Chang Y and Moore PS (1996) Seroprevalence of Kaposi's sarcoma-associated herpes virus antibodies among Americans, Italians and Ugandans with and without Kaposi's sarcoma. *Nature Med* **2**: 925–928

Geddes M, Franceschi S, Balzi D, Arniani S, Gafà L and Zanetti R (1995) Birthplace and classic Kaposi's sarcoma in Italy. *J Natl Cancer Inst* **87**: 1015–1017

McCullagh P and Nelder JA (1989) *Generalized Linear Models,* 2nd edition. Chapman & Hall: Cambridge

Safai B, Mikè V, Giraldo G, Beth E and Good RA (1980) Association of Kaposi's sarcoma with second primary malignancies. Possible etiopathogenic implications. *Cancer* **45**: 1472–1479

Salvaggio JE and Sullivan KA (1992) Environmental chemicals and the immune system. In *Environmental and Occupational Medicine*, Rom WN. (ed.), pp. 69–88. Little, Brown and Company: Boston

Schmauz R, Mugerwa JW and Wright DH (1990) The distribution of non-Burkitt, non-Hodgkin's lymphomas in Uganda in relation to malarial endemicity. *Int J Cancer* **45**: 650–653

Smith AG, Bustamante CI and Wood C (1993) Disseminated cutaneous and vascular invasion by *Fusarium moniliforme* in a fatal case of acute lymphocytic leukemia. *Mycopathologia* **122**: 15–20

Statistics and Epidemiology Research Corporation (1988) EGRET. SERC: Seattle

Veglia KS and Marks VJ (1987) Fusarium as a pathogen. A case report of Fusarium sepsis and review of the literature. *J Am Ac Dermatol* **16**: 260–263

Warren CPW (1992) Health and safety in the grain industry. In *Environmental and Occupational Medicine*, Rom WN (ed.), pp. 381–391. Little, Brown and Company: Boston

© Cancer Research Campaign 1997